# Congenital Heart Disease: The Crossroads of Genetics, Epigenetics and Environment

**DOI:** 10.2174/1389202915666140716175634

**Published:** 2014-10

**Authors:** Cecilia Vecoli, Silvia Pulignani, Ilenia Foffa, Maria Grazia Andreassi

**Affiliations:** 1 CNR, Institute of Clinical Physiology, Pisa, Italy; 2 CNR, Institute of Clinical Physiology, Massa, Italy;; 3 Fondazione Toscana G.Monasterio, Massa, Italy

**Keywords:** Congenital heart disease, CHD, Genetics, Epigenetics, Environment, Point mutations, Methylation, microRNA.

## Abstract

Congenital heart diseases (CHDs) are recognized as the most common type of birth malformations. Although recent advances in pre- and neonatal diagnosis as well as in surgical procedures have reduced the morbidity and mortality for many CHD, the etiology for CHD remains undefined. In non-syndromic and isolated (without a familial history or a Mendelian inheritance) forms of CHDs, a multifactorial pathogenesis with interplay between inherited and non-inherited causes is recognized. In this paper, we discuss the current knowledge of the potential molecular mechanisms, mediating abnormal cardiac development in non-syndromic and isolated CHD, including mutations in cardiac transcription factors, the role of somatic mutations and epigenetic alterations as well as the influence of gene-environment interactions. In the near future, the advent of high-throughput genomic technologies with the integration of system biology will expand our understanding of isolated, non-syndromic CHDs for their prevention, early diagnosis and therapy.

## INTRODUCTION

Congenital heart disease (CHD) is the most common type of congenital malformation affecting 1.35 million newborns each year. Nowadays, excluding the infectious causes of death, CHD represents the first non-syndromic, sporadic defect in the first year of life [1, 2]. However, the worldwide prevalence of all CHD may exceed these estimates. Undoubtedly, CHD defines a large set of structural and functional defects that arise during embryogenesis, including mild lesions which are clinically quiescent for decades such as bicuspid aortic valve (with a prevalence of 0.5-0.9% [3]). A recent meta-analysis showed that the prevalence of CHD varies across countries and continents, with a higher prevalence in Europe than in the North America [4]. The recent advances in pre- and neonatal diagnosis as well as in surgical procedures have reduced morbidity and mortality for many CHDs, although substantial risks remain for the most severe forms of CHD [5-7]. Today, more than 75% of CHD children who survive the first year of life, including those with complex malformations, will live to adulthood [8]. As a result, an increasing cardiovascular disease population, with a high risk of transmitting the disease, will reach the reproductive age [7]. Such a population poses physiological and socioeconomic challenges not only for patients and their families but also for all of the society. Thus, a greater understanding of CHD’s genetic and molecular mechanisms is fundamental, in order to improve diagnosis and genetic counseling.

Multiple and sophisticated experimental animal models, produced by perturbing selected genes that work in cardiac developmental pathways (such as myocytes, neural crest, proepicardium, or endocardium specification, differentiations and morphogenesis), have provided important insights into CHDs’ molecular mechanisms. In humans, the basis of these cardiac perturbations is more complex and remains poorly understood. Moreover, most of the cases of CHDs are diagnosed as non-syndromic, sporadic defects. Thus, neither a family history nor a clear Mendelian inheritance of the disease may be recognized. In these cases, a multifactorial pathogenesis with a strong interplay between inherited and non-inherited causes is recognized [8, 9].

The purpose of this paper is to discuss the current knowledge of the molecular mechanisms mediating abnormal cardiac development in non-syndromic and isolated CHD, including mutations in the components of the cardiac transcription factor (TF) network, the potential role of somatic mutations and epigenetic alterations as well as the influence of gene*-*environment interactions.

## GENETIC CAUSES OF ISOLATED CHD

**Fig. (1):**
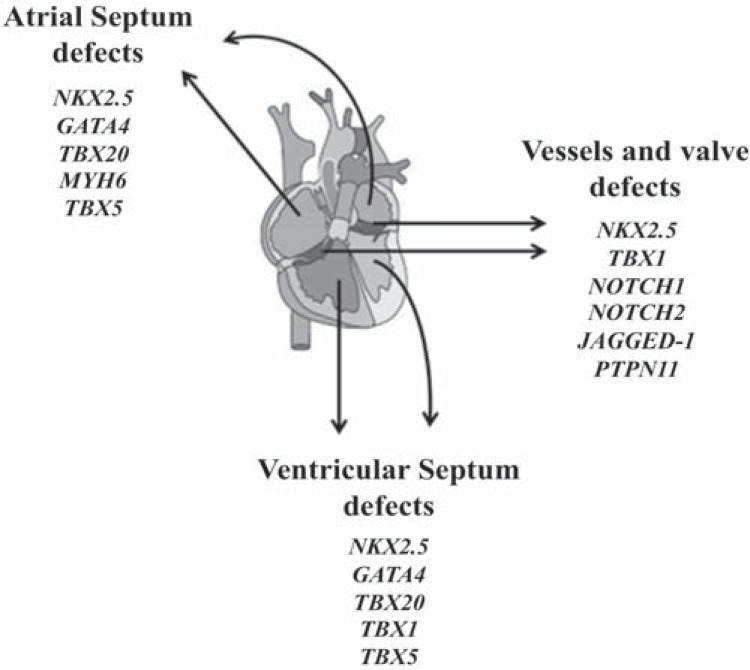
Example of how three kind of congenital heart defects (atrial septum defects, ventricular septum defects and vessels and valve defects) are associated with gene mutations in TFs and signaling molecules.

The list of gene point mutations that cause isolated, non-syndromic CHD is rapidly expanding [10, 11] (Table **1**). A specific class of genes involved in transcriptional controls, the so-called TFs, has been identified as the major player in cardiac development (Fig. **1**) [12, 13].

**Fig. (2):**
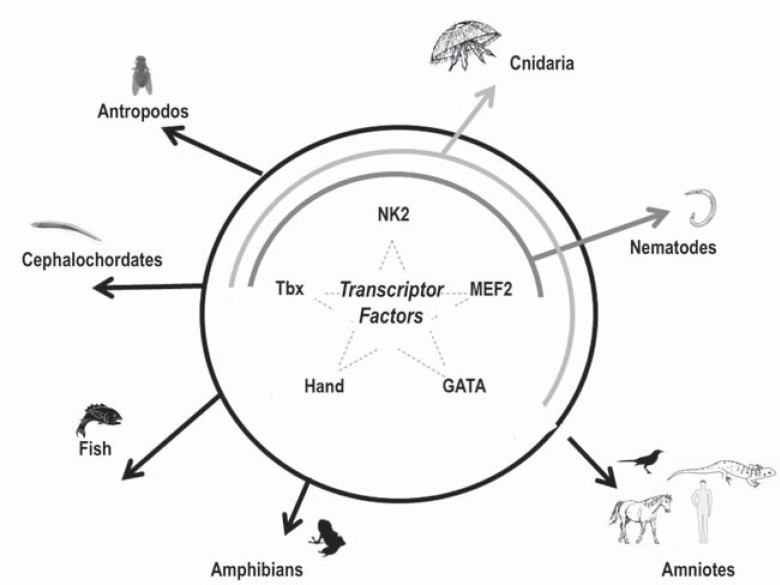
The complexity of development varies among organisms despite the conserved core of transcription factor genes. In the figure, the most important evolutionarily conserved transcription factors (NK2, MEF2, GATA, Tbx, and Hand) known to control cardiac human cell fates are reported in different classes of organisms. In Nematodes NK2, MEF2, Tbx has been found (internal line); in Cnidaria NK2, MEF2, GATA and Tbx have been found (middle line) while in Antropodos, Cephalochordates, Fish, Amphibians, and Amniotes all the TFs (NK2, MEF2, GATA, and Tbx) are conserved (external circle line).

TFs minutely drive specific events in the complex process of cardiogenesis regulating each other’s expression, in order to stabilize and reinforce the cardiac gene program. Indeed, cardiovascular structure development requires the generation of diverse cell types, including cardiomyocytes,

conduction system, smooth muscle, valvular and endothelial cells, in a defined spatio-temporal coordinated manner. The availability of experimental models in which specific genes are inactivated or over expressed has allowed the identification of several TFs highly conserved in organisms ranging from insects to higher vertebrates, and their involvement in heart development [14-16] (Fig. **2**). The interconnections between numerous TFs, their downstream target genes, and upstream signaling pathways activate the expression of genes that control cardiac cell fates and morphogenesis of cardiac structures deriving from the early embryonic heart field [8, 15]. 

For instance, Hiroi *et al.* [16] provided evidence that Tbx5 (T-box 5) is associated with Nkx2-5 (NK2 homeobox 5) and synergistically promotes cardiomyocyte differentiation. Indeed, by using the yeast two-hybrid system, these authors showed that Tbx5 and Nkx2-5, synergistically binding the promoter of Nppa (cardiac-specific natriuretic peptide precursor type A), are able to activate this gene inducing cardiac development. Animal experimental studies showed that embryos of mice knockout for Gata4 (GATA binding protein 4) gene died just before the heart tube fusion [17, 18]. Gata4 heterozygote mice are phenotypically normal, but mice homozygous for a hypomorphic Gata4 allele show a spectrum of embryonic cardiac abnormalities such as atrioventricular septal defects [19]. Already in 1997, Durocher *et al.* showed that GATA-4 and Nkx2-5 interact physically and synergistically to activate cardiac transcription [20]. A study of large pedigrees with familial CHD led to the discovery of a strict interaction between Gata4 and Tbx5 [21]. Specifically, identified a missense mutation in Gata4 (that disrupted a highly conserved glycine residue) has been identified, able to disrupt the Gata4-Tbx5 interaction, maintaining the Gata4-Nkx2.5 interaction. Since in previous studies Tbx5 has been shown to interact with Nkx2.5, this study supported evidence that all the three transcription factors could physically interact *in vitro* [16]. Altogether, these papers suggest a functional convergence of different pathways involved in the cardiac development and any mutation in any of these three genes can result in a cardiac defect. Consistent with this, also mutations in MYH6, a specific target of GATA4 and TBX5, have been associated with atrial septal defects [22]. Furthermore, other interaction partners have been identified (for major details see the reviews [23-26]) and it has been observed that the loss of function of different TFs has dramatic consequences in cardiovascular development.

In humans, the clinical spectrum of malformations which arise from single TF mutations is extremely broad and the genotype-phenotype associations is complex; a given structural defect can be caused by more than one gene, because of TFs network. Mutations in GATA4 gene can cause not only inherited septation defects [27], but also atrioventricular canal defects [28]. Mutations in NKX2.5 were identified as a genetic cause of several CHD phenotypes (e.g. atrial /ventricular septal defects, coarctation of aorta) including electrophysiological alterations similar to TBX5 gene [29]. However, the frequency of mutations in TF genes in sporadic, non-syndromic cases of CHD is very low (0- 3%) [30-32]. Thus, several authors have suggested the involvement of other genes and molecular mechanisms.

Mutations in a variety of genes that encode molecules participant in development signaling pathways have been shown to occur in different forms of sporadic congenital cardiac defects. As example, Notch signaling is a well-known vital pathway for the cardiac development including cardiac fate determination, patterning of the primitive heart and cardiac morphogenesis. NOTCH1 mutations have been associated with dominantly inherited BAV [33-36], and, more recently, also with left ventricular outflow tract malformations [36, 37]. Additionally, mutations in cardiac structural proteins have been identified as monogenic cause of CHD. Mutations in elastin ELN gene have been found in same sporadic cases of supravalvolar aortic stenosis [38]. Similarly, rare missense mutations in MYH6 gene (cardiac muscle protein-coding gene) can cause sporadic atrial septal defects [39, 40].

Rare (≤1% population frequency) copy number variants (CNVs), that are large deletions or amplifications of DNA segments arising mainly from inappropriate recombination, seem to be the cause of 5%-10% of sporadic, non-syndromic CHDs [41]. Large de novo CNVs (present in probands but absent in both parents) have been reported in tetralogy of Fallot [42], left-sided lesions [43] and other isolated cases of CHDs [44-46]. Some CNVs have been identified in previously classified CHD genes. For instance, recurrent CNVs in GATA4 gene have been found in CHD cases [47], and CNVs at chromosome 20p12.2 and 9q34.3 impact members of Notch signaling pathway (JAG1 and NOTCH1) [48]. Last year, in an elegant study of exome sequencing performed in parent-offspring trios, Zaidi *et al.* found a marked excess of *de novo* mutations in genes involved in normal heart development [49]. The mutations also impacted genes causing Mendelian CHDs, although a variable expressivity and a broader phenotypic spectrum resulted from mutations at known disease loci. These findings implicate *de novo* point mutations in several hundred genes that collectively contribute to ~10% of severe CHD.

## SOMATIC MUTATIONS AND CHD

In 2004, Reamon-Buettner *et al*. [50] showed “*for the first time that malformed hearts are affected by multiple somatic NKX2–5 mutations*” paving the way to the somatic mutations hypothesis as a novel genetic mechanism for CHDs [50]. Somatic mutations can arise in distinct cell lineages in embryonic development or during postnatal life. Furthermore, the mutation analysis is usually performed in constitutional DNA, whereas the mutations might be somatic and limited to only a subset of cells or tissues, leading to mosaicism, with two or more genetically distinct cell lines within the same organism. The result may be a milder disease phenotype or can unmask the expression of a mutation that would otherwise be lethal to the embryo. Somatic mutations are mostly recognized as a key factor in the genetics of cancer and a multitude of somatic mutations from cancer genomes have been reported [51]. Idiopathic forms of human disease are rarely considered having a genetic origin but, in some cases, somatic mutations were found to be the cause of different sporadic diseases [52, 53]. In CHDs, somatic mutations have been reported in NKX2.5, TBX5, GATA4 and HAND1 in DNA extracted from tissues of malformed hearts, fixed in formalin and stored for more than 20 years at the University of Leipzig [54-56]. However, later studies in fresh frozen cardiac tissues did not confirm the abundance of somatic mutations previously published [57-59]. However, the identification of cardiac-specific somatic mutations of the connexin 40 (Cx40) and connexin 43 (Cx43) genes in sporadic atrial fibrillation strongly supports that somatic or tissue-specific genetic variants can cause cardiac defects [60, 61]. Similarly, a more recent study identified two novel heterozygous mutations of GATA6 (p.G367X and p.G394C) in the malformed heart tissues of patients with tetralogy of Fallot. The mutant alleles were absent in the cardiac tissues of patients with rheumatic heart disease and in the peripheral blood samples of the participants [62]. From these evidences, we cannot completely exclude low-level of somatic changes in the pathogenesis of CHD (likely undetectable by using conventional sequencing) although it is assumed that they are very minor contributors.

## EPIGENETICS AND CHD

## DNA Methylation and Histone Modifications

The genotype of most cells of a given organism is the same (with the exception of gametes and the cells of the immune system), while cellular phenotypes and functions differ radically. This discrepancy can be due to differential epigenetic regulation. As proposed by Conrad Waddington in the 1950s,* “An epigenetic trait is a stably heritable phenotype resulting from changes in a chromosome without alterations in the DNA sequence”* that can involve the heritability of a phenotype, passed on through either mitosis or meiosis.

Thus, these DNA or chromatin modifications can arise during both cell differentiation and embryonic morphogenesis, or during the mitotic divisions of a cell and play a critical role in the regulation of different genomic functions. DNA methylation was the first epigenetic mechanism discovered and is involved in a variety of biological process including embryonic development, X-chromosome inactivation and genomic imprinting [63]. Altered methylation status has been widely described in carcinogenesis as a major mechanism of gene silencing [64].

A similar mechanism of gene silencing has been recently shown in patients with sporadic tetralogy of Fallot, where an aberrant methylation status of NKX2-5 and HAND1 (heart and neural crest derivatives expressed transcript 1) has been found [65]. Indeed, the authors demonstrated that this abnormal methylation is negatively correlated with the mRNA expression of NKX2-5 and HAND1 in cardiac tissue. This evidence suggests that DNA methylation changes can contribute to a transcription down-regulation of two genes essential for the heart development [65].

Furthermore, a case-control study showed an association between high levels of methylation biomarkers and complex syndromic CHD in very young children [66]. In a different study, a global hypomethylation state in mothers and in their children with Down’s syndrome and cardiac defects suggested the heritability of global methylation state as well as a possible role in the segregation of chromosomes. Indeed, accumulating evidence strongly suggested that environmental exposures 'in utero' can influence the so-called 'epigenome', resulting in cardiac defects or diseases developed later in life. In addition, abnormal DNA methylation patterns in sperm may be another critical mechanism, resulting in impaired fertility and compromising spermatogenesis [67].

Chromatin remodeling and histone modification have substantial roles in activating or silencing gene expression [68, 69]. It is known that the function of TFs is strongly associated with the status of the chromatin. The major modulators of the chromatin structure are the actions of ATP-dependent chromatin remodeling complexes such as BAF chromatin remodeling complexes and chemical modification of histones [69]. The BAF chromatin remodeling complexes are polymorphic assemblies of more components able to modify DNA–nucleosome interactions thereby remodeling chromatin [70].

In the developing heart, BAF60c, a subunit of the SWI/SNF-like BAF chromatin remodeling complex (also called Smarcd3), is highly expressed. *In vitro* BAF60c mediates the interaction of cardiac TFs (GATA4, NKX2.5, and TBX5) with the SWI/SNF complex ATPase Brg1, potentiating their transcriptional activation [71]. 

More recently, Baf60c has emerged as a rate-limiting factor in *de novo* activation of the cardiac program, in concert with Gata4 and Tbx5 [72]. How TFs interact with BAF60c and more generally how they induce cardiogenesis are incompletely understood so far.

From a molecular point of view, histone modification is considered a central epigenetic mark like methylation but, to date, little is known about its roles in embryonic development. In a study, Miller *et al.* demonstrated that T-box factors are able to alter the state of chromatin at their target genes [73].

Specifically, the authors showed that “T-box family possesses both the capability to alleviate repressive as well as establish permissive epigenetic states through their interaction with H3K27-demethylase and H3K4-methyltransferase activities, respectively” [73].

The effect of this mechanism on heart development is not known. Conversely, it is known that several mutations disrupting the interactions between the T-box domain and these epigenetic-modifying proteins are associated to human genetic diseases [73]. Between the chromatin-modifying enzymes, histone deacetylases (HDACs) play a major role since, by removing acetyl groups from histone tails, they compact the chromatin and repress transcription [74]. In an experimental model of conditional knockout mice, Montgomery *et al.* showed that the global deletion of HDAC1 led to an earlier embryonic death than the global deletion of HDAC2. Indeed, HDCA2 knockout mice survived until the perinatal period but died early with several cardiac defects. Conversely, the cardiac-specific deletion of one of two genes did not induce a specific phenotype, whereas if both genes were lacking at cardiac levels, cardiac arrhythmias, dilation as well as the up-regulation skeletal muscle-specific contractile proteins and calcium channels caused a neonatal death. A key regulatory role of histone modification is therefore conceivable in controlling the expression of genes codifying for protein involved in cardiac morphogenesis, growth, and contractility during development and also throughout life [75].

## MicroRNA

In recent years, the discovery of small non-coding RNAs known as microRNAs (miRNAs, non-protein-coding small molecules of RNA, 20-22 nucleotides) has provided evidence for crucial post-translational mechanisms that are classified as new epigenetic markers. The specific biological roles of most miRNAs are still unknown. Several studies have showed their control of several cellular pathophysiological pathways that play a significant role in the pathogenesis of many disease states. 

Notably, studies showed that miRNAs mainly hybridize with specific sequence in the 3'UTR of specific targets regulating different processes of animal development and physiology [76, 77].

In 2005, miR-1 was identified to play a key role in cardiomyocyte differentiation [78-80]. miR-1, specifically expressed in cardiac precursor cells, is a direct transcriptional target of muscle differentiation regulators. During cardiogenesis, an excess of miR-1 decreased the number of proliferating ventricular cardiomyocytes modulating regulatory proteins involved in the differentiation/proliferation balance control. In addition, overexpression of miR-1 decreased the level of Hand2 protein without changing its mRNA level, suggesting that Hand2 is a target of miR-1 during heart development [78]. Deletion of miR-1-2 results in heart defects that include ventricular septum defects leading to defects in conduction system and in increased cardiomyocyte proliferation [80]. Recently, by sequencing the GATA4 gene, we found several variants in its 3’UTR region [81]. The presence of genetic variants such as single nucleotide polymorphisms (SNPs) in 3’UTR may affect the bond strength of a specific miRNA, so that one allele may reduce or eliminate the binding [82-85], modulating gene expression. By using a computational approach followed by an *in vitro* functional analysis, we found that +1521C > G in the 3’UTR of GATA4 gene is a functional variant that can influence the susceptibility to CHD by affecting the post-transcriptional control by miRNAs [81].

Recent studies have also shown that miRNA profile can change in relation to the exposure to several environmental factors such as air pollution, metals and cigarette smoking [86, 87]. It has been hypothesized that environmental chemicals induce miRNA expression alterations via increasing oxidative stress and/or triggering inflammatory processes. Whether and how environmental exposure affects miRNA expression remains to be determined.

## THE BIOLOGICAL IMPACT OF ENVIRONMENTAL FACTORS

Little is known regarding risk factors for CHDs, although numerous, easily modifiable ‘‘environmental exposure’’ factors (referring to any non-genetic factor and more specifically to the fetal-placental-maternal environment) have been shown to play a major role in the development of CHDs. The proportion of CHDs potential prevention through changes in the fetal environment is unknown, but around 30% has been estimated for some defects [88]. Up to now, few data on modifiable risk factors do not allow to develop strategies to assist couples in making lifestyle choices to reduce the probability of having a child with a CHD [9].

Since the critical period for cardiac development is between 2 and 7 weeks of gestational age, the major impact of environmental risk factors is limited to parental conditions and environmental exposures during the periconceptional period (generally defined as 3 months before pregnancy through the first trimester of pregnancy) [9, 89].

Exposure to toxic agents (such as smoking, alcohol consumption or exposure to chemicals) [9] during the pre-conceptional period plays a relevant role in the risk of CHD because the DNA in each human cell (and so also in spermatozoa and in oocytes) can be affected by exposure to environmental toxicants. Gametic DNA mutations preceding conception can induce miscarriage, death or congenital defects.

Potential environmental risk factors known to increase the incidence of CHD are maternal exposure, such as maternal smoking, alcohol consumption, rubella virus infection, and maternal exposure to chemicals at work during the 3-month pre-conception period or during the pregnancy [9]. The use of some drugs such as barbituric acid or chemotherapy agents, exposure to organic solvents and pesticides and the onset of gestational diabetes have been identified as potential environmental risk factors for CHD [90, 91]. Conversely, it is known that multivitamin use contributes to the prevention of CHD [92, 93], while the use of folate antagonists, dihydrofolate reductase inhibitors or antiepileptics increases CHD risk [94, 95]. 

The effect of paternal environmental exposure as a risk factor for CHD has scarcely been evaluated, but experimental and more recent epidemiological evidences highlight its major role. In addition to toxicant compounds that can be adsorbed by sperm and introduced directly into the egg at fertilization, any contaminants (such as pesticides, metals, nicotine and its metabolites or aromatic hydrocarbons) in semen are transmitted (through ejaculation) to a woman. Once in the woman’s body, the contaminant may reach and affect the current pregnancy or remain to influence future pregnancies [96, 97]. The effects of paternal exposures on offspring health have been documented in animal models [98]. In humans, there is evidence of paternally mediated Mendelian genetic defects, chromosomal aberrations, and aneuploid sperm [96]. However, further well-designed studies are necessary to explain the biological mechanisms of exposure–mediated abnormal cardiac development. 

## GENE-ENVIRONMENTAL INTERACTIONS

Nowadays it is clear that people have different susceptibilities to the effects of toxic agent exposure [99]. There is a growing number of allelic variants (genetic polymorphisms) of genes encoding enzymes involved in biotransformation/detoxification of toxicants and in DNA repair, that may be responsible for a different susceptibility toward environmental toxicants [100]. Indeed, the personal genetic background significantly contributes to predisposing a person to an illness during environmental exposure. Thus, new research approaches focusing on studying the individual (genetic) susceptibility to environmental toxicants may be a critical component of molecular epidemiology, especially for congenital defects [101, 102]. 

For instance, a strong interaction between polymorphisms in genes involved in detoxification pathways and maternal cigarette smoking has been reported on orofacial defects [101]. Glutathione S-transferase (GST) GSTM1 and GSTT1 are fundamental enzymes in the detoxification system. In a recent study, we showed that specific and common genetic variants in GSTM1 and GSTT1 genes can modify a person's risk of toxicant exposure-induced disease [103]. In this study, we clearly showed that parental exposure to toxicants (such as paternal smoking) increased the risk of having children with CHDs, proving evidence of the influence of environmental factors for congenital malformations.

In the last decade, among the non-genetic causes of CHD, a major role seems to be played by assisted reproductive techniques although the risk varies with the method of assisted reproductive technique [104]. The authors speculate that this may not only be due to the reproductive technology but also due to the underlying reason for infertility of the couple strongly related to the environmental conditions.

In a recent review focused on epigenetics in cardiovascular disease, Ordovás and Smith [105] indicated as a key point that “*the prenatal environment can induce changes in gene expression that are independent of the DNA sequence and arise as a result of epigenetic mechanisms*”. As previously mentioned, accumulating evidence suggests that the epigenome is particularly susceptible to environmental factors during embryogenesis, when the DNA synthesis rate is high and the DNA methylation pattern is established [105, 106]. In 2011, Chowdhury *et al.* [107] provides the first evidence of association between maternal gene-specific DNA methylation and CHD by a case-control study of genome-wide maternal DNA methylation. They identified more than 400 CpG sites differentially methylated between cases and controls involving more than 400 genes. Interestingly, the list of differentially methylated genes included 14 miRNA sites while the differentially methylated genes were involved in fetal development [107]. 

The importance of these results is emphasized since more studies show that the environment-dependent epigenetic gene regulation may persist transgenerationally [108, 109].

## NEW STRATEGIES FOR DEFINING THE GENETIC ARCHITECTURE OF CHD

Due to the expected multifactorial nature of isolated CHD, the identification of causal genes through direct sequencing of candidate target genes has not been very successful. High-throughput whole genome, exome sequencing or target resequencing has opened a new era of DNA analyses in which thousands of variations can be screened. Similarly, by using both microarray platforms and high-throughput RNA sequencing, also differences in gene expression (transcriptomics) can be assessed [110]. System biology which integrates complex datasets of cogent pathways obtained from animal models to humans that operate in multidimensional spaces, provides new strategies for elucidating the pathogenesis of CHDs. Using bioinformatics and computational algorithms, able to integrate different datasets from different studies of CHD in both humans and model organisms, system biology will allow us to explore systematically the relationship between CHD (genetic and non-genetic) risk factors and responses [111-114].

## CONCLUSION

CHDs represent a considerable burden of personal suffering and societal cost. Although enormous progress has been made in understanding the complexity of cardiac development, the fundamental etiology for congenital heart defects remains undefined. 

From the data collected in this review, it is clear that the genetic causes identified so far as well as the only environmental exposure are unable to account for the population prevalence of sporadic CHD. 

At present, too many questions remain open regarding the disease. Future integrative studies including different causative aspects are needed to provide a better understanding of the molecular basis of CHD. This integrative revision of CHD genetics, epigenetics and exposure risk factors suggests a complex pattern of interaction that modulates critical biological systems during cardiogenesis. A single specific mechanism cannot explain the complex etiology of CHD and the phenotypic heterogeneity observed in CHD patients. As speculated by Lage *et al.* [2012]: “ …*malformations arise from a multidimensional combinatorial space of perhaps tens or hundreds of sequence and structural variants that may perturb several different members of large developmental networks in a single patient. The individual genetic risk factors might be very rare, and the specific combination of risk factors in many patients unique, which could account for the considerable phenotypic heterogeneity observed in CHD patients with identical mutations in established CHD genes” *[113]. Thus, a *two-hit model* is plausible where inherited genetic variants predispose the heart but require a second (genetic or environmental) hit to cause the disease. Large longitudinal studies in which robust analysis of genome, exome, and mRNA expression (reflecting the genetic and epigenetic transcription level) together with a deep evaluation of environmental exposure (as well as lifestyle) would be the potential to test this *two-hit model* hypothesis. This approach could aid to find the functional convergence of risk factors in highly polygenic disorders such as CHD, suggesting new prevention strategies and diagnostic methods based on genetics. The power of more cost-effective high-throughput sequencing approaches (from exome to transcriptome through genome) and the access to data from large international efforts will provide the opportunity to carry out more accurate multi-disciplinary studies. International networks will also be crucial for providing comprehensive study of genetic, epigenetic, and environment crossroads to achieve greater power to disentangle the complexity of isolated, non-syndromic CHDs.

## Figures and Tables

**Table 1.** Transcription factors implicate in cardiac morphogenesis.

Gene	Cardiac Phenotypes	OMIM
ANKRD1	*Total Anomalous Pulmonary Venous Return*	609599
CITED2	*Ventricular Septal Defect* * Atrial Septal Defect*	602937
GATA4	*Atrial Septal Defect* * Atrioventricular Septal Defect*	600576
*FOXH1*	*Tetralogy Of Fallot* * Congenital Heart Malformation*	603621
GATA6	*Persistent Truncus Arterious Pulmonary Valve*	601656
HAND2	*Tetralogy Of Fallot*	602407
HEY2	*Tricuspid Atresia*	604674
IRX4	*Ventricular Septal Defect*	606199
NKX2-5	*Atrial Septal Defect- Atrioventricular-Block* * Tetralogy Of Fallot* * Hypoplastic Left Heart,* * Interrupted Aortic Arch* * Heterotaxy* * Transposition Of The Great Arteries* * Double Outlet Right Ventricle* * Ventricular Septal Defect*	600584
NKX2-6	*Persistent Truncus Arterious*	611770
TBX1	*Ventricular Septal Defect* * Interrupted Aortic Arch*	602054
TBX5	*Atrial Septal Defect, Ventricular Septal Defect, Atrioventricular Septal Defect*	601620
TBX20	*Atrial Septal Defect, Coa* * Ventricular Septal Defect* * Patent Ductus Arterious* * Hypoplastic Left Ventricle* * Atrial Septal Defect*	606061
TFAP2B	*Patent Arterial Duct*	601601
ZFPM2	*Tetralogy Of Fallot* * Double Outlet Right Ventricle*	603693
ZIC3	*Heterotaxy* * Transposition Of The Great Arteries* * Atrial Septal Defect* * Pulmonary Valve*	300265

## References

[1] Hoffman JL, Kaplan S (2002). The incidence of congenital heart disease.. J. Am. Coll. Cardiol..

[2] Posch MG, Perrot A, Berger F, Ozcelik C (2010). Molecular genetics of congenital atrial septal defects.. Clin. Res. Cardiol..

[3] Nistri S, Basso C, Marzari C, Mormino P, Thiene G (2005). Frequency of bicuspid aortic valve in young male conscripts by echocardiogram.. Am. J. Cardiol..

[4] van der Linde D, Konings EE, Slager MA, Witsenburg M, Helbing WA, Takkenberg JJ, Roos-Hesselink JW (2011). Birth prevalence of congenital heart disease worldwide a systematic review and meta-analysis.. J. Am. Coll. Cardiol..

[5] Bonnet D, Coltri A, Butera G, Fermont L, Le Bidois J, Kachaner J, Sidi D (1999). Detection of transposition of the great arteries in fetuses reduces neonatal morbidity and mortality.. Circulation.

[6] Khoshnood B, De Vigan C, Vodovar V, Goujard J, Lhomme A, Bonnet D, Goffinet F (2005). Trends in prenatal diagnosis, pregnancy termination, and perinatal mortality of newborns with congenital heart disease in France 1983–2000 a population-based evaluation.. Pediatrics.

[7] Deanfield J, Thaulow E, Warnes C, Webb G, Kolbel F, Hoffman A, Sorenson K, Kaemmer H, Thilen U, Bink-Boelkens M, Iserin L, Daliento L, Silove E, Redington A, Vouhe P, Priori S, Alonso MA, Blanc JJ, Budaj A, Cowie M, Deckers J, Fernandez Burgos E, Lekakis J, Lindahl B, Mazzotta G, Morais J, Oto A, Smiseth O, Trappe HJ, Klein W, Blömstrom-Lundqvist C, de Backer G, Hradec J, Mazzotta G, Parkhomenko A, Presbitero P, Torbicki. A (2003). Task Force on the Management of Grown Up Congenital Heart Disease European Society of Cardiology,.ESC Committee for Practice Guidelines. The Task Force on the management of grown-up congenital heart disease of the European Society of Cardiology management of grown-up congenital heart disease.. Eur. Heart J..

[8] Pierpont ME, Basson  C.T., Basson D.W. , Gelb  B.D., Giglia  TM, Goldmuntz  E., McGee  G, Sable  C.A., Srivastava  D, Webb CL (2007). Genetic basis for congenital heart defects current knowledge a scientific statement from the American Heart Association Congenital Cardiac Defects Committee, Council on Cardiovascular Disease in the Young endorsed by the American Academy of Pediatrics.. Circulation.

[9] Jenkins KJ, Correa A, Feinstein JA, Botto L, Britt AE, Daniels SR, Elixson M, Warnes CA, Webb CL (2007). Noninherited risk factors and congenital cardiovascular defects current knowledge a scientific statement from the American Heart Association Council on Cardiovascular Dis-ease in the Young endorsed by the American Academy of Pediatrics.. Circulation.

[10] Wessels MW, Willems PJ (2010). Genetic factors in non-syndromic congenital heart malformations.. Clin. Genet..

[11] Fahed AC, Gelb BD, Seidman JG, Seidman CE (2013). Genetics of congenital heart disease the glass half empty.. Circ. Res..

[12] Srivastava D, Olson EN (2000). A genetic blueprint for cardiac development.. NAture.

[13] Olson EN (2006). Gene regulatory networks in the evolution and development of the heart.. ScIence.

[14] Biben C, Weber R, Kesteven S, Stanley E, McDonald L, Elliott DA, Barnett L, Köentgen F, Robb L, Feneley M, Harvey RP (2000). Cardiac septal and valvular dysmorphogenesis in mice heterozygous for mutations in the homeobox gene Nkx2-5.. Circ. Res..

[15] Laforest B, Nemer M (2011). GATA5 interacts with GATA4 and GATA6 in outflow tract development.. Dev Biol..

[16] Hiroi Y, Kudoh S, Monzen K, Ikeda Y, Yazaki Y, Nagai R, Komuro I (2001). Tbx5 associates with Nkx2-5 and synergistically promotes cardiomyocyte differentiation.. Nature Genet..

[17] Molkentin J D, Lin Q, Duncan SA, Olson EN (1997). Re-quirement of the transcription factor GATA4 for heart tube formation and ventral morphogenesis.. Genes Dev..

[18] Kuo CT, Morrisey EE, Anandappa R, Sigrist K, Lu MM, Parmacek MS, Soudais C, Leiden JM (1997). GATA4 transcription factor is required for ventral morphogenesis and heart tube formation.. Genes Dev..

[19] Pu WT, Ishiwata T, Juraszek AL, Ma Q, Izumo S (2004). GATA4 is a dosage-sensitive regulator of cardiac morpho-genesis.. Dev. Biol..

[20] Durocher D, Charron F, Warren R, Schwarz RJ, Nemer M (1997). The cardiac transcription factors Nkx2-5 and GATA-4 are mutual cofactors.. EMBO J..

[21] Garg V, Kathiriya I S, Barnes R, Schluterman M K, King I N, Butler CA, Rothrock CR, Eapen RS, Hirayama-Yamada K, Joo K, Matsuoka R, Cohen JC, Srivastava D (2003). GATA4 mutations cause human congenital heart defects and reveal an interaction with TBX5.. Nature..

[22] Ching Y H, Ghosh T K, Cross S J, Packham E A, Honeyman L, Loughna S, Robinson TE, Dearlove AM, Ribas G, Bonser AJ, Thomas NR, Scotter AJ, Caves LS, Tyrrell GP, Newbury-Ecob RA, Munnich A, Bonnet D, Brook JD (2005). Mutation in myosin heavy chain 6 causes atrial septal defect.. Nat. Genet..

[23] Garg V (2006). Insight into the genetic basis of congenital heart disease.. Cell. Mol. Life Sci..

[24] Ransom J, Srivastava D (2007). The genetics of cardiac birth defects.. Semin. Cell. Dev. Biol..

[25] Bruneau BG (2008). The developmental genetics of congenital heart disease.. Nature.

[26] Huang JB, Liu YL, Sun PW, Lv XD, Du M, Fan XM (2010). Molecular mechanisms of congenital heart disease.. Cardio-vasc Pathol..

[27] Chen Y, Han ZQ, Yan WD, Tang CZ, Xie JY, Chen H, Hu DY (2010). A novel mutation in GATA4 gene associated with dominant inherited familial atrial septal defect.. J. Thorac. Cardiovasc. Surg..

[28] Moskowitz IP, Wang J, Peterson MA, Pu WT, Mackinnon AC, Oxburgh L, Chu GC, Sarkar M, Berul C, Smoot L, Robertson EJ, Schwartz R, Seidman JG, Seidman CE (2011). Transcription factor genes Smad4 and Gata4 cooperatively regulate cardiac valve development.. Proc. Natl. Acad. Sci. USA..

[29] Schott JJ, Benson DW, Basson CT, Pease W, Silberbach GM, Moak JP, Maron BJ, Seidman CE, Seidman JG (1998). Congenital heart disease caused by mutations in the transcription factor NKX2-5.. Science.

[30] Posch MG, Perrot A, Schmitt K, Mittelhaus S, Esenwein EM, Stiller B, Geier C, Dietz R, Gessner R, Ozcelik C, Berger F (2008). Mutations in GATA4, NKX25, CRELD1, and BMP4 are infrequently found in patients with congenital cardiac septal defects.. Am. J. Med. Genet..

[31] Pulignani S, Foffa I, Cresci M, Vittorini S, Ait-Ali L, Andreassi MG (2011). Genetic screening of GATA4 and NKX2. mutations in hereditary congenital heart defects 5 familial cases.. Recenti Prog. Med..

[32] Blue GM, Kirk EP, Sholler GF, Harvey RP, Winlaw DS (2012). Congenital heart disease current knowledge about causes and inheritance.. Med. J. Aust..

[33] Garg V, Muth AN, Ransom JF, Schluterman MK,  Barnes R, King IN, Grossfeld PD, Srivastava D (2005). Mutations in NOTCH1 cause aortic valve disease.. Nature.

[34] Mohamed SA, Aherrahrou Z, Liptau H, Erasmi AW, Hagemann C, Wrobel S, Borzym K, Schunkert H, Sievers HH, Erdmann J (2006). Novel missense mutations (p.596M and p.P1797H) in NOTCH1 in patients with bicuspid aortic valve.. Biochem. Biophys. Res. Commun..

[35] McKellar SH, Tester DJ, Yagubyan M, Majumdar R, Ackerman  J, Sundt TM (2007). Novel NOTCH1 mutations in patients with bicuspid aortic valve disease and thoracic aor-tic aneurysms.. J. Thorac. Cardiovasc. Surg..

[36] Foffa I, Ait Ali L, Panesi P, Mariani M, Festa P, Botto N, Vecoli C, Andreassi MG (2013). Sequencing of NOTCH1, GATA5, TGFBR1 and TGFBR2 genes in familial cases of bicuspid aortic valve.. BMC Med. Genet..

[37] McBride KL, Riley MF, Zender GA, Fitzgerald-Butt SM, Towbin JA, Belmont JW, Cole SE (2008). NOTCH1 mutations in individuals with left ventricular outflow tract malformations reduce ligand-induced signaling.. Hum. Mol. Genet..

[38] Metcalfe K, Rucka AK, Smoot L, Hofstadler G, Tuzler G, McKeown P, Siu V, Rauch A, Dean J, Dennis N, Ellis I, Reardon W, Cytrynbaum C, Osborne L, Yates JR, Read AP, Donnai D, Tassabehji M (2000). Elastin mutational spectrum in supravalvular aortic stenosis.. Eur. J. Hum. Genet..

[39] Matsson H, Eason J, Bookwalter CS, Klar J, Gustavsson P, Sunnegårdh J, Enell H, Jonzon A, Vikkula M, Gutierrez I, Granados-Riveron J, Pope M, Bu'Lock F, Cox J, Robinson TE,  Song F, Brook DJ, Marston S:, Trybus KM, Dahl N (2008). Alpha-cardiac actin mutations produce atrial septal defects.. Hum. Mol. Genet..

[40] Granados-Riveron JT, Ghosh TK, Pope M, Bu'Lock F, Thornborough C, Eason J, Kirk EP, Fatkin D, Feneley MP, Harvey RP, Armour JA, David Brook J (2010). Alpha-cardiac myosin heavy chain (MYH6) mutations affecting myofibril formation are associated with congenital heart de-fects.. Hum. Mol. Genet..

[41] Soemedi R, Wilson IJ, Bentham J, Darlay R, Töpf A, Zelenika D, Cosgrove C, Setchfield K, Thornborough C, Granados-Riveron J, Blue GM, Breckpot J, Hellens S, Zwolinkski S, Glen E, Mamasoula C, Rahman TJ, Hall D, Rauch A, Devriendt K, Gewillig M, O' Sullivan J, Winlaw DS, Bu'Lock F, Brook JD, Bhattacharya S, Lathrop M, Santibanez-Koref M, Cordell HJ, Goodship JA, Keavney BD (2012). Contribution of global rare copy-number variants to the risk of sporadic congenital heart disease.. Am. J. Hum. Genet..

[42] Silversides CK, Lionel AC, Costain G, Merico D, Migita O, Liu B, Yuen T, Rickaby J, Thiruvahindrapuram B, Marshall CR, Scherer SW, Bassett AS (2012). Rare copy number variations in adults with tetralogy of Fallot implicate novel risk gene pathways.. PLoS Genet..

[43] Hitz MP, Lemieux-Perreault LP, Marshall C, Feroz-Zada Y, Davies R, Yang SW, Lionel AC, D'Amours G, Lemyre E, Cullum R, Bigras JL, Thibeault M, Chetaille P, Montpetit A, Khairy P, Overduin B, Klaassen S, Hoodless P, Awadalla P, Hussin J, Idaghdour Y, Nemer M, Stewart AF, Boerkoel C, Scherer SW, Richter A, Dubé MP, Andelfinger G (2012). Rare copy number variants contribute to congenital left-sided heart disease.. PLoS Genet..

[44] Payne AR, Chang SW, Koenig SN, Zinn AR, Garg V (2012). Submicroscopic chromosomal copy number variations identified in children with hypoplastic left heart syndrome.. Pediatr. Cardiol..

[45] Cooper GM, Coe BP, Girirajan S, Rosenfeld JA, Vu TH, Baker C, Williams C, Stalker H, Hamid R, Hannig V, Abdel-Hamid H, Bader P, McCracken E, Niyazov D, Leppig K, Thiese H, Hummel M, Alexander N, Gorski J, Kussmann J, Shashi V, Johnson K, Rehder C, Ballif BC, Shaffer LG, Eichler EE (2011). A copy number variation morbidity map of developmental delay.. Nat. Genet..

[46] Erdogan F, Larsen LA, Zhang L, Tümer Z, Tommerup N, Chen W, Jacobsen JR, Schubert M, Jurkatis J, Tzschach A, Ropers HH, Ullmann R (2008). High frequency of submicroscopic genomic aberrations detected by tiling path array comparative genome hybridisation in patients with isolated congenital heart disease.. J. Med. Genet..

[47] Tomita-Mitchell A, Maslen CL, Morris CD, Garg V, Goldmuntz E (2007). GATA4 sequence variants in patients with con-genital heart disease.. J. Med. Genet..

[48] Greenway SC, Pereira AC, Lin JC, DePalma SR, Israel SJ, Mesquita SM, Ergul E, Conta JH, Korn JM, McCarroll SA, Gorham JM, Gabriel S, Altshuler DM, Quintanilla-Dieck Mde L, Artunduaga MA, Eavey RD, Plenge RM, Shadick NA, Weinblatt ME, De Jager PL, Hafler DA, Breitbart RE, Seidman JG, Seidman CE (2009). De novo copy number variants identify new genes and loci in isolated sporadic tetralogy of Fallot.. Nat. Genet..

[49] Zaidi S, Choi M, Wakimoto H, Ma L, Jiang J, Overton JD, Romano-Adesman A, Bjornson RD, Breitbart RE, Brown KK, Carriero NJ, Cheung YH, Deanfield J, DePalma S, Fakhro KA, Glessner J, Hakonarson H, Italia MJ, Kaltman JR, Kaski J, Kim R, Kline JK, Lee T, Leipzig J, Lopez A, Mane SM, Mitchell LE, Newburger JW, Parfenov M, Pe'er I, Porter G, Roberts AE, Sachidanandam R, Sanders SJ, Seiden HS, State MW, Subramanian S, Tikhonova IR, Wang W, Warburton D, White PS, Williams IA, Zhao H, Seidman JG, Brueckner M, Chung WK, Gelb BD, Goldmuntz E, Seidman CE, Lifton RP (2013). De novo mutations in histone-modifying genes in congenital heart disease.. Nature.

[50] Reamon-Buettner SM, Hecker H, Spanel-Borowski K, Craatz S, Kuenzel E, Borlak J (2004). Novel NKX2-5 mutations in diseased heart tissues of patients with cardiac malformations.. Am. J. Pathol..

[51] Stratton  MR., Campbell  PJ, Futreal PA (2009). The cancer genome.. Nature.

[52] Erickson RP (2003). Somatic gene mutation and human disease other than cancer.. Mutat. Res..

[53] Puck JM, Straus SE (2004). Somatic mutations - not just for cancer anymore.. N. Engl. J. Med..

[54] Reamon-Buettner SM, Borlak J (2006). Somatic mutations in cardiac malformations.. J. Med. Genet..

[55] Reamon-Buettner SM, Cho SH, Borlak J (2007). Mutations in the 3'-untraslated region of GATA4 as molecular hotspots for congenital heart disease [CHD].. BMC Med. Genet..

[56] Reamon-Buettner SM, Borlak J (2004). Somatic NKX2-5 mutations as a novel mechanism of disease in complex con-genital heart disease.. J. Med. Genet..

[57] Draus Jr JM, Hauck MA, Goetsch M, Austin III EH, Tomita-Mitchell A, Mitchell ME (2009). Investigation of somatic NKX2-5 mutations in congenital heart disease.. J. Med. Genet..

[58] Wang J, Lu Y, Chen H, Yin M, Yu T, Fu Q (2011). Investigation of somatic NKX25, GATA4 and HAND1 muta-tions in patients with tetralogy of Fallot.. Path..

[59] Salazar M, Consoli F, Villegas V, Caicedo V, Maddaloni V, Daniele P, Caianiello G, Pachón S, Nuñez F, Limongelli G, Pacileo G, Marino B, Bernal J E, De Luca A, Dallapiccola B (2011). Search of somatic GATA4 and NKX2. gene mutations in sporadic septal heart defects.. Eur. J. Med. Genet..

[60] Gollob MH, Jones DL, Krahn AD, Danis L, Gong XQ, Shao Q, Liu X, Veinot JP, Tang AS, Stewart AF, Tesson F, Klein GJ, Yee R, Skanes AC, Guiraudon GM, Ebihara L, Bai D (2006). Somatic mutations in the connexin 40 gene [GJA5] in atrial fibrillation.. N. Engl. J. Med..

[61] Thibodeau IL, Xu J, Li Q, Liu G, Lam K, Veinot JP, Birnie DH, Jones DL, Krahn AD, Lemery R, Nicholson BJ, Gollob MH (2010). Paradigm of genetic mosaicism and lone atrial fibrillation physiological characterization of a connexin 43-deletion mutant identified from atrial tissue.. Circulation.

[62] Huang RT, Xue S, Xu YJ, Yang YQ (2013). Somatic mutations in the GATA6 gene underlie sporadic tetralogy of Fallot.. Int. J. Mol. Med..

[63] Bird A (2002). DNA methylation patterns and epigenetic memory.. Genes Dev..

[64] Esteller M (2007). Epigenetic gene silencing in cancer the DNA hypermethylome.. Hum. Mol. Genet..

[65] Sheng W, Qian Y, Wang H, Ma X, Zhang P, Diao L, An Q, Chen L, Ma D, Huang G (2013). DNA methylation status of NKX2-5, GATA4 and HAND1 in patients with tetralogy of fallot.. BMC Med. Genomics..

[66] Obermann-Bort SA, Van Driel LM, Helbing WA, de Jonge R, Wildhagen MF, Steegers EA, Steegers-Theunissen RP (2011). Congenital heart defects and biomarkers of methylation in children a case-control study.. Eur. J. Clin. Invest..

[67] Aitken RJ, De Iuliis GN (2007). Origins and consequences of DNA damage in male germ cells.. Reprod. Biomed. Online.

[68] Ohtani K, Vlachojannis GJ, Koyanagi M, Boeckel JN, Urbich C, Farcas R, Bonig H, Marquez VE, Zeiher AM, Dimmeler S (2011). Epigenetic regulation of endothelial lineage committed genes in pro-angiogenic hematopoietic and endothelial progenitor cells.. Circ. Res..

[69] Bruneau BG (2010). Chromatin remodeling in heart development.. Curr. Opin. Genet. Dev..

[70] Ho L, Crabtree GR (2010). Chromatin remodelling during development.. Nature.

[71] Lickert H, Takeuchi JK, von Both I, Walls JR, McAuliffe F, Adamson SL, Henkelman RM, Wrana JL, Rossant J, Bruneau BG (2004). Baf60c is essential for function of BAF chromatin remodeling complexes in heart development.. Nature.

[72] Takeuchi JK, Bruneau BG (2009). Directed transdifferentiation of mouse mesoderm to heart tissue by defined factors.. Nature.

[73] Miller SA, Huang AC, Miazgowicz MM, Brassil MM, Weinmann AS (2008). Coordinated but physically separable interac-tion with H3K27-demethylase and H3K4-methyltransferase activities are required for T-box protein-mediated activation of developmental gene expression.. Genes Dev..

[74] Grozinger CM, Schreiber SL (2002). Deacetylase enzymes biological functions and the use of small-molecule inhibitors.. Chem. Biol..

[75] Montgomery RL, Davis CA, Potthoff MJ, Haberland M, Fielitz J, Qi X, Hill JA, Richardson JA, Olson EN (2007). Histone deacetylases 1 and 2 redundantly regulate cardiac morphogenesis, growth, and contractility.. enes Dev..

[76] Conne B, Stutz A, Vassalli JD (2000). The 3’ untraslated region of messenger RNA A molecular ‘hotspot’ for pathologyκ. Nat. Med..

[77] Lee CT, Risom T, Strauss WM ( 2006). MicroRNAs in mammalian development.. Birth Defects Res.C.; Embryo. Today..

[78] Zhao Y, Samal E, Srivastava D (2005). Serum sponse factor regulates a muscle-specific microRNA that targets Hand2 during cardiogenesis.. Nature.

[79] Kwon C, Han Z, Olson EN, Srivastava D (2005). MicroRNA1 influences cardiac differentiation in Drosophila and regulates Notch signaling.. Proc. Natl. Acad. Sci. USA..

[80] Zhao Y, Ransom JF, Li A, Vedantham V, von Drehle M, Muth AN, Tsuchihashi T, McManus MT, Schwartz RJ, Srivastava D (2007). Dysregulation of cardiogenesis, cardiac conduction, and cell cycle in mice lacking miRNA-1-2.. Cell..

[81] Sabina S, Pulignani S, Rizzo M, Cresci M, Vecoli C, Foffa I, Ait-Ali L, Pitto L, Andreassi MG (2013). Germline hereditary. somatic mutations and microRNAs targeting-SNPs in congenital heart defects.. J. Mol. Cell. Cardiol..

[82] Abelson JF, Kwan KY, O’Roak BJ, Baek DY, Stillman AA, Morgan TM, Mathews CA, Pauls DL, Rasin MR, Gunel M, Davis NR, Ercan-Sencicek AG, Guez DH, Spertus JA, Leckman JF, Dure 4th LS, Kurlan R, Singer HS, Gilbert DL, Farhi A, Louvi A, Lifton RP, Sestan N, State MW (2005). Sequence variants in SLITRK1 are associated with Tourette’s syndrome.. Science.

[83] Clop A, Marcq F, Takeda H, Pirottin D, Tordoir X, Bibe B, Bouix J, Caiment F, Elsen JM, Eychenne F, Larzul C, Laville E, Meish F, Milenkovic D, Tobin J, Charlier C, Georges M (2006). A mutation creating a potential illegitimate microRNA target site in the myostatin gene affects muscularity in sheep.. Nat. Genet..

[84] Sethupathy P, Collins FS (2008). MicroRNA target site polymorphisms and human disease.. Trends Genet..

[85] Wang G, van der Walt JM, Mayhew G, Li YJ, Zuchner S, Scott WK, Martin ER, Vance JM (2008). Variation in the miRNA-433 binding site of FGF20 confers risk for Parkinson disease by overexpression of alpha-synuclein.. Am. J. Hum. Genet..

[86] Hou L, Wang D, Baccarelli A (2011). Environmental chemicals and microRNAs.. Mutation Res..

[87] Bollati V, Marinelli B, Apostoli P, Bonzini M, Nordio F, Hoxha M, Pegoraro V, Motta V, Tarantini L, Cantone L, Schwartz J, Bertazzi PA, Baccarelli A (2010). Exposure to metal-rich particulate matter modifies the expression of candidate microRNAs in peripheral blood leukocytes.. Environ. Health Perspect..

[88] Wilson PD, Loffredo CA, Correa-Villasenor A, Ferencz C (1998). Attributable fraction for cardiac malformations.. Am. J. Epidemiol..

[89] Srivastava D (2001). Genetic assembly of the heart implications for congenital heart disease.. Annu. Rev. Physiol..

[90] Loffredo CA, Wilson PD, Ferencz C (2001). Maternal diabetes an independent risk factor for major cardiovascular malformation with increased mortality of affected infants.. Teratology..

[91] Bracken MB (1986). Drug use in pregnancy and congenital heart disease in offspring.. N. Engl. J. Med..

[92] Botto LD, Mulinare J, Erickson JD (2000). Occurrence of congenital heart defects in relation to maternal multivitamin use.. Am. J. Epidemiol..

[93] Shaw GM, O'Malley CD, Wasserman CR, Tolarova MM, Lammer EJ (1995). Maternal periconceptional use of multivitamins and reduced risk for conotruncal heart defects and limb deficiencies among offspring.. Am. J. Med. Genet. A..

[94] Hernandez-Diaz S, Werler MM, Walker AM, Mitchell AA (2000). Folic acid antagonists during pregnancy and the risk of birth defects.. N. Engl. J. Med..

[95] Meijer WM, de Walle HE, Kerstjens-Frederikse WS, de Jong-van den Berg LT (2005). Folic acid sensitive birth defects in association with intrauterine exposure to folic acid antagonists.. Reprod. Toxicol..

[96] Chapin RE, Robbins WA, Schieve LA, Sweeney AM, Tabacova SA, Tomashek KM ( 2004). Off a good start the influence of pre- and periconceptional exposures. parental fertiity.and nutrition on children s health.. Environ. Health. Perspect..

[97] Gianicolo EA, Cresci M, Ait-Ali L, Foffa I, Andreassi MG (2010). Smoking and congenital heart disease the epidemiologi-cal and biological link.. Curr. Pharm. Des..

[98] Hales BF, Robaire. B (2001). Paternal exposure to drugs and environmental chemicals effects on progeny outcome.. J. Androl..

[99] Olden K, Wilson S (2000). Environmental health and genomics visions and implications.. Nat. Rev. Genet..

[100] Olshan AF, Shaw GM, Millikan RC, Laurent C, Finnell RH (2005). Polymorphisms in DNA repair genes as risk factors for spina bifida and orofacial clefts.. Am. J. Med. Genet. A..

[101] Kelada SN, Eaton DL, Wang SS, Rothman NR, Khourym MJ (2003). The role of genetic polymorphisms in environmental health.. Environ. Health. Perspect..

[102] Bolt HM, Their R (2006). Relevance of the deletion polymorphisms of the glutathione S-transferases GSTT1 and GSTM1 in pharmacology and toxicology.. Curr. Drug. Metab..

[103] Cresci M, Foffa I, Ait-Ali L, Pulignani S, Gianicolo EA, Botto N, Picano E, Andreassi MG (2011). Maternal and Paternal Environmental Risk Factors. Metabolizing GSTM1 and GSTT1 Polymorph sms.and Congenital Heart Disease.. Am. J. Cardiol..

[104] Tararbit K, Houyel L, Bonnet D, De Vigan C, Lelong N, Goffinet F, Khoshnood B (2011). Risk of congenital heart defects associated with assisted reproductive technologies a population-based evaluation.. Eur. Heart. J..

[105] Ordovás JM, Smith CE (2010). Epigenetics and cardiovascular disease.. Nat. Rev. Cardiol..

[106] Dolinoy DC, Weidman JR, Jirtle RL (2007). Epigenetic gene regulation linking early developmental environment to adult disease.. Reprod. Toxicol..

[107] Chowdhury S, Erickson SW, MacLeod SL, Cleves MA, Hu P, Karim MA, Hobbs CA (2011). Maternal genome-wide DNA methylation patterns and congenital heart defects.. PLoS One..

[108] Lane N, Dean W, Erhardt S, Hajkova P, Surani A, Walter J, Reik W (2003). Resistance of IAPs to methylation reprogramming may provide a mechanism for epigenetic inheritance in the mouse.. Genesis..

[109] Morgan HD, Sutherland HG, Martin DI, Whitelaw E (1999). Epigenetic inheritance at the agouti locus in the mouse.. Nat. Genet..

[110] Hawkins RD, Hon GC, Ren B (2010). Next-generation genomics an integrative approach.. Nat. Rev. Genet..

[111] Chen X, Jorgenson E, Cheung ST (2009). New tools for functional genomic analysis.. Drug. Discov. Today..

[112] Lage K, Greenway SC, Rosenfeld JA, Wakimoto H, Gorham JM, Segrè AV, Roberts AE, Smoot LB, Pu WT, Pereira AC, Mesquita SM, Tommerup N, Brunak S, Ballif BC, Shaffer LG, Donahoe PK, Daly MJ, Seidman JG, Seidman CE, Larsen LA (2012). Genetic and envi-ronmental risk factors in congenital heart disease functionally converge in protein networks driving heart development.. Proc. Natl. Acad. Sci. USA..

[113] Lage K, Møllgård K, Greenway S, Wakimoto H, Gorham JM, Workman CT, Bendsen E, Hansen NT, Rigina O, Roque FS, Wiese C, Christoffels VM, Roberts AE, Smoot LB, Pu WT, Donahoe PK, Tommerup N, Brunak S, Seidman CE, Seidman JG, Larsen LA (2010). Dis-secting spatio-temporal protein networks driving human heart development and related disorders.. Mol. Syst. Biol..

[114] He D, Liu ZP, Chen L (2011). Identification of dysfunctional modules and disease genes in congenital heart disease by a network-based approach.. BMC Genomics..

